# RNA design: update on computational frameworks and programs for inverse RNA folding

**DOI:** 10.1093/bib/bbag327

**Published:** 2026-06-19

**Authors:** Sumit Mukherjee, Rami Zakh, Alexander Churkin, Danny Barash

**Affiliations:** Institute for Interdisciplinary Computational Science, Ben-Gurion University, David Ben-Gurion Blvd. 1, Be’er-Sheva, 8410501, Israel; Cancer Data Science Laboratory, Center for Cancer Research, National Cancer Institute, National Institutes of Health (NIH), Bethesda, 20892, MD, United States; Institute for Interdisciplinary Computational Science, Ben-Gurion University, David Ben-Gurion Blvd. 1, Be’er-Sheva, 8410501, Israel; Department of Software Engineering, Sami Shamoon College of Engineering, 56 Bialik St. Be’er-Sheva, 8410802, Israel; Department of Software Engineering, Sami Shamoon College of Engineering, 56 Bialik St. Be’er-Sheva, 8410802, Israel; Institute for Interdisciplinary Computational Science, Ben-Gurion University, David Ben-Gurion Blvd. 1, Be’er-Sheva, 8410501, Israel

**Keywords:** RNA design, inverse RNA folding

## Abstract

Programs and computational frameworks for predicting RNA sequences with desired folding properties are continually being developed and expanded. A decade has passed since they were last reviewed in this journal, and this brief review provides an update to the review published at that time. Given a target secondary structure, these programs aim to predict RNA sequences that fold into the desired structure while satisfying various constraints. This procedure is known as inverse RNA folding. Traditionally, inverse RNA folding has been used to design optimized RNAs with favorable properties. This updated review covers some of the most widely used freeware programs developed for this purpose over the past decade. RNAinverse, part of the Vienna RNA package, was the first program devised to address the inverse RNA folding problem, and many subsequent programs were described in the earlier review. Some of the most important computational frameworks are the Infrared framework and DesiRNA. In addition, RNA design capabilities have been incorporated into the RNAstructure package, while NUPACK, as well as MoiRNAiFold, MODENA, incaRNAfbinv, and related tools have undergone recent updates. A variety of strategies have also emerged to address the problem of 3D RNA design and RNA–RNA interactions. The various programs mentioned employ distinct approaches, ranging from replica exchange Monte Carlo to constraint satisfaction, as well as Boltzmann sampling and machine learning approaches. Machine learning methods are being developed for emerging applications in biotechnology such as messenger RNA(mRNA) design and CRISPR guide RNA (gRNA) design. This brief review examines these programs and provides a timely update.

## Introduction

RNA design is solving an inverse RNA folding problem, in which the goal is to design RNA sequences that fold into a given RNA secondary structure. Inverse RNA folding was introduced in the early 1990s in Vienna [1]. Over the years, RNA design has been generalized to include, for example, the design of RNA sequences that fold into a given tertiary structure, as well as further generalizations, including approaches that emphasize a variety of constraints. They are highly relevant to the design of artificial constructs of a variety of functional RNAs [2–6], such as in the emerging fields of RNA synthetic biology [7, 8] and RNA nanostructure [9]. Some examples for the use of RNA design in emerging fields can be found at [10–14]. RNA inverse folding is also used in biological evolution studies, where the sequence/structure relationship in RNA secondary structure is a well-known model for studying genotype/phenotype maps [15]. Recent applications such as mRNA design are further discussed in the *Discussion* Section.

The approach to solve the inverse RNA folding problem is by stochastic optimization. It relies on the solution of the direct problem of predicting the RNA secondary structure from sequence using software that is also commonly available in RNA folding prediction web servers, e.g. the RNAfold server [16, 17] or mfold/UNAFold [18, 19] as well as RNAstructure [20], by performing energy minimization with thermodynamic parameters [21, 22]. In principle, other programs can also be used to solve the direct problem, for example CentroidFold [23], or others that employ machine learning (ML)/ artificial intelligence (AI) although these are less accurate to-date for RNA secondary structure prediction when compared to biophysical models because of insufficient training data with biases [24, 25]. Thus, classical RNA design programs operate as follows. Initially, a seed sequence is chosen, and a local search strategy is then used to mutate the seed and repeatedly apply the direct problem of the RNA folding prediction by energy minimization. After that, in the vicinity of the seed sequence, a designed sequence is located with desired folding properties according to an objective function in the initial optimization problem formulation. In the mid-2000s, algorithmic improvements relevant to this approach that was pioneered in Vienna’s RNAinverse [1] have been put forward in INFO-RNA [26], RNA-SSD [27] and NUPACK:Design [28, 29]. Alternative approaches to the adaptive random walk [1] and the stochastic local-search [26, 27] also include genetic algorithms (belonging to the class of evolutionary algorithms) [30, 31], constraint programming [32] and ant-colony optimization [33]. Another powerful idea for efficiency improvement that began to emerge is weighted sampling [34], which was added to a fragment-based design approach that generalized the inverse RNA folding problem [35] and culminated in incaRNAfbinv [36] that provides more flexibility in the design as compared to the aforementioned methods. The benefit of this generalization was illustrated in the previous review on RNA design in this journal [37], along with other computational frameworks that were described. To avoid repetition, in what follows new developments in the past decade will be described that follows RNAdesign [38] (mentioned last in [37]) for designing RNA sequences that fold into multiple target structures and EternaBot [39] that was developed to design a sequence based on rules by Eterna players in the Eterna project [40].

The problem of tertiary structure design of RNAs has witnessed some major developments in the past decade since RNA-redesign [41] that solved the local problem of fixed backbone 3D design and was described in the previous review. The RNAMake design algorithm was put forth in [42], as well as gRNAde [43] and R3Design [44] employing state-of-the-art machine learning models. It appears that the success of machine learning models in the tertiary structure design relative to biophysical models is in general considerably better than in the secondary structure design. In recent years, traditional tertiary structure folding prediction methods have also been complementing design tasks, as with RNAComposer [45] in [46] and utilizing SimRNA [47] in [48], while the latter group of SimRNA came with the new design methodology of DesiRNA [49] that will be described herein in more detail. Another important problem is that of sequence design for RNA–RNA interactions, with the latest design approach named RRIdesign described in [50]. Finally, there were some notable theoretical advances such as that designing RNA secondary structures is NP-hard [51] and more recent advances showing that RNA inverse folding can be solved in linear time for structures without isolated stacks or base pairs [52] and the interesting topic of undesignable motifs [53].

## New computational frameworks

In the past decade, a large collection of new software tools has been introduced. While many of them are important for various specific tasks, and only some are listed in this brief updated review because of a lack of space, here we single out two new software developments (or computational frameworks) that were especially developed for general RNA design, without undermining the importance of all other new software. The first one is the Infrared framework [54, 55], and the second one is DesiRNA [49]. These two computational frameworks were selected because they are more general than the rest of the newly developed programs, allowing significantly more features and capabilities, and they are substantially more time efficient. Because RNA design is an inverse problem, the methods to solve this problem are time-consuming and with high computational demand. The Infrared framework solves it by algorithmic efficiencies. For example, by using tree decomposition, this framework handles RNA design tasks by solving them with dynamic programming algorithms, achieving efficiency that is polynomial (and often linear) in the number of variables. It also uses fixed-parameter tractable (FPT) algorithms, sampling, and other ingredients to gain efficiency. DesiRNA takes a different approach and among all other Monte-Carlo methods, it is highly efficient because it uses parallel tempering. As mentioned, its success in solving all 100 puzzles in the Eterna 100 benchmark within 24 h is impressive.

**Figure 1:**
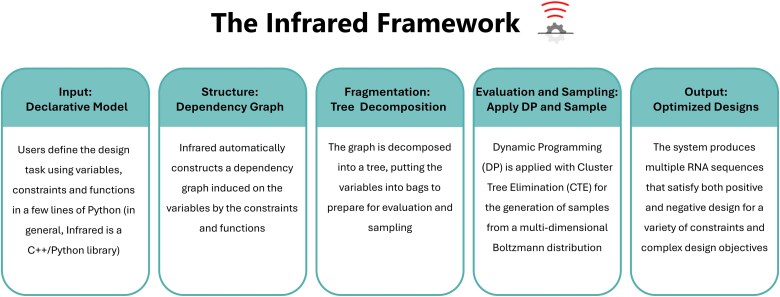
The infrared framework.

The Infrared framework is a generic framework for efficient combinatorial sampling, with the RNA design problem formalized as a constraint satisfaction problem with designed objectives described as a set of constraints and a set of weighted functions. This past decade has witnessed a noticeable advancement in RNA design tools that addressed a variety of design challenges, for example negative design problems (in which the target structure(s) have the best energies among all structures, avoiding good energies for all other structures) in addition to positive design problems, multi-structure design, and complex constraints. This progress started with a tool called RNAblueprint [56], then came RNARedprint [57, 58], and throughout the general Infrared framework was developed [54, 55]. It offers sophisticated algorithms along with user-friendly interfaces, in our context for designing RNA sequences with control over the RNA molecule’s structural and functional properties. Figure 1 attempts to provide an overview of this general framework although many details are omitted for simplicity and the interested reader is encouraged to examine the online manual referred to in the references.

This framework has the advantage that it is highly flexible (e.g. can target multiple structures simultaneously, with a variety of constraints that can be easily added) and it is fast and efficient by using advanced computational methods. An example of a recent tool that was developed with this framework is the RRIdesign for advanced RNA–RNA interactions in the sequence design [50], and a very recent application for the rational design of mechanically active RNAs was worked out in [48]. It is especially recommended for experienced users who are faced with extensive design problems.

**Figure 2:**
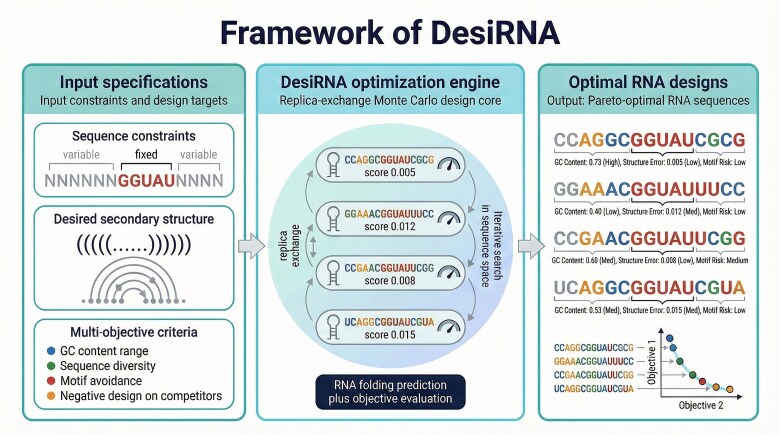
Framework of DesiRNA.

Another computational tool that is quite general in its capabilities, but in a different way relative to the Infrared framework, is DesiRNA [49]. It uses replica-exchange Monte Carlo, or simulated annealing with parallel tempering, for solving the RNA design problem. The generality here is that it can also account for oligomerization in the design and, versatile design capabilities also include two-strand RNA complexes, RNAs with alternative structures, RNA with pseudoknots, GC content, and positive and negative designs (these capabilities overlap with the Infrared framework capabilities). It boasts exceptional performance in solving EteRNA100 challenges, solving all 100 design problems in under 24 h in which 90 were solved under one minute on a standard computer platform. The DesiRNA computational framework with the design process partitioned into three primary stages is illustrated in Fig. 2.

The first stage of the Input Specifications is depicted in the left-most panel. The user defines designed targets, including 5 bp sequence constraints with a central fixed motif (GGUAU) and flanking variable regions (N). The target secondary structure is provided via Vienna’s dot-bracket notation (e.g. ((((( … …)))))) and visualized with arc diagrams. Optimization is guided by multi-objective criteria such as GC content, sequence diversity, and motif avoidance. The second stage of the Optimization Engine is depicted in the middle panel. The core search utilizes a replica-exchange Monte Carlo design core. This engine performs an iterative search in sequence space, utilizing parallel replicas to ensure robust exploration. Each candidate undergoes RNA folding prediction and objective evaluation to generate a fitness score. The third stage of Optimal RNA designs is depicted in the right-most panel. The output provides a set of Pareto-optimal sequences. These represent the best possible trade-offs between competing objectives (e.g. maximizing structural stability versus achieving a specific GC target). The Pareto front graph visualizes this relationship, where each colored point corresponds to a unique designed sequence. DesiRNA is especially recommended for practitioners who are interested in experimenting with an additional layer beyond a simple RNA design.

## Discussion

**Figure 3:**
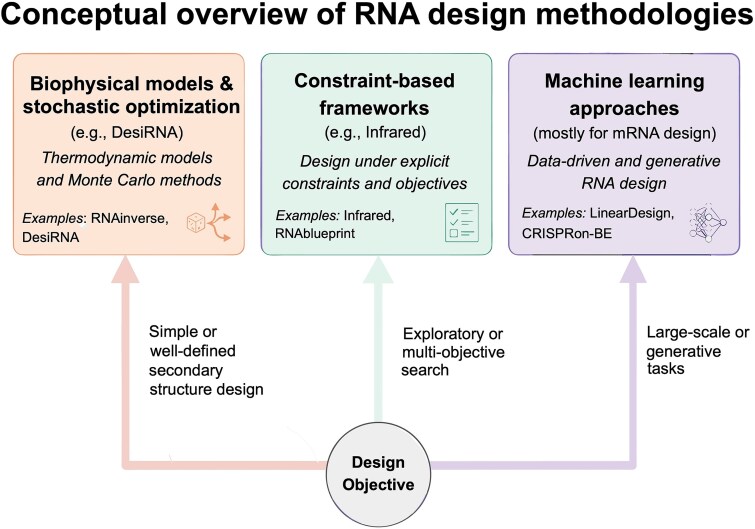
Conceptual overview of RNA design methodologies. RNA design approaches can be broadly categorized into thermodynamic models and stochastic optimization methods, constraint-based frameworks, and emerging machine learning-based approaches. The figure highlights the underlying principles of each class and their typical applications across different design objectives, providing a high-level conceptual framework for relating specific tools or computational frameworks to their broader methodological context.

Aside of the two new computational frameworks that were mentioned in the previous sections, there were many advanced programs developed for more specific uses. Here we briefly group some of them, although other important programs are not listed. To better contextualize this growing diversity of tools, it is useful to consider how these approaches relate to broader classes of RNA design methodologies. As summarized in Fig. 3, RNA design strategies can be broadly categorized into biophysical models coupled with stochastic optimization methods, constraint-based frameworks, and emerging machine learning–based approaches. These classes are not mutually exclusive and can be combined depending on the problem at hand. For example, in mRNA vaccine design, machine learning-based approaches could be advantageous, whereas in many other applications effective solutions can be obtained using the other methodological classes. In addition, machine learning techniques can complement thermodynamic models, for instance by enhancing pre-processing. The figure explicitly includes DesiRNA and the Infrared framework.

In the group of programs that were developed within well-known packages, one should refer to the new RNA design capability within RNAstructure [20] described in [59] via ‘Design’ for structured RNA sequence design and ‘Orega’ for unstructured RNA sequence design. Also, the established NUPACK [29] has been developed to an all-new NUPACK 4 [60]. MoiRNAiFold, an extension of RNAiFold [32], was introduced in [61] and further exemplified in [62]. Other notable extensions that were put forth from previous approaches include incaRNAfbinv 2.0 [63], an extension of MODENA [31] for riboswitches described in [64], and the newest SIMARD [65].

In this update review, we do not consider benchmarking, as this was recently done comprehensively in [66] and on an adjacent problem of neural nucleic acids design algorithms [67] that is more specific. In the aforementioned work, an alternative to the Eterna 100 benchmark was put forth, and also in this new benchmark DesiRNA was found to achieve best performance in many of the cases examined. As was noted in the previous section, DesiRNA was able to solve all EteRNA 100 challenges. Another program that recently managed to succeed in it is Montparnasse [68], with a more specialized algorithm that comes from the game playing field. It should be noted that improved efficiency ideas for Monte Carlo schemes of this sort include the use of machine learning as a pre-processing step before RNA design, which was demonstrated in [69] with data from Rfam [70]. Other more specialized data, such as from RiboD [71] for more specific purposes like riboswitches can also be thought of, as well as using synthetic data. It should be noted that in this approach machine learning is only performed at the pre-processing stage, as in [72] for the identification of intrinsic transcription terminators, but RNA folding prediction using biophysical models is still used as the core method of choice for the reasons explained in [24]. Even without machine learning, constraint programming was used for the identification of IRES-like structural subdomains [73] and the design of highly active double-pseudoknotted ribozymes [74], fragment-based RNA design [36] has been shown beneficial as a pre-processing step before Infernal [75] for riboswitch identification in [76], and with the same strategy in preliminary results [77] that preceded [78] for the identification of HDV RNA editing elements. Machine learning can further enhance these identification attempts as the first pre-processing component in the pipeline, while still retaining thermodynamic models at the core as the second and main pre-processing component of RNA design. Thus, various components in a pipeline can serve different purposes, with machine learning models enhancing rather than replacing thermodynamic models. Constraint-based frameworks, such as the advanced Infrared framework, can also be integrated when needed, and all combinations are possible depending on the objective.

Many new programs were developed using machine learning models instead of biophysical models for the inverse RNA folding problem from secondary structure to sequence, despite the problematic issues described in [24, 25]. Examples of such works are LEARNA [79] and DRAG [80]. For more specific applications in RNA design, machine learning has been used successfully for CRISPR gRNA design in [81, 82] (CRISPRon/off, CRISPRon-BE) and broadly reviewed in [83]. It has also been applied to molecular RNA switch design using restricted Boltzmann machines [84]. In addition, it has been used in RaptGen [85] and RfamGen [86] for generative aptamer discovery and RNA family design, respectively, as reviewed in [87]. Some additional specific applications in RNA design that utilize machine learning can be found in [88] and throughout the literature.

Finally, the problem of mRNA design is emerging, with mRNA folding recently reviewed in [89] and a breakthrough achieved in [90] using LinearDesign. Recent programs for mRNA design include SamplingDesign [91], JAX-RNAfold [92], which uses differentiable folding [93], EnsembleDesign [94], mRNA-LM and mRNA-GPT [95, 96], GEMORNA [97], mRNAdesigner [98], mRNAarchitect [99], GARDN [100], and RiboTree [101, 102], among others. The Infrared framework has demonstrated broad utility in the rational design of functional RNAs, as exemplified in [48], and represents a general constraint-based design paradigm that may be transferable to mRNA design. Integrating this framework with complementary approaches, including thermodynamic RNA structure prediction models and, where appropriate, machine learning based methods, may improve mRNA design performance relative to individual methods. While machine-learning-based methods may offer advantages in usability for specific applications such as mRNA vaccine design, their performance can be limited by the quality and diversity of available training data.

**Figure 4:**
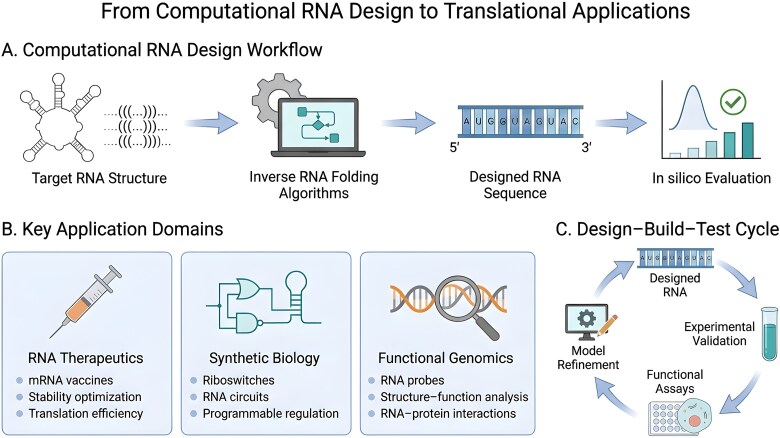
From computational RNA design to functional and translational applications. Computational RNA design workflows enable the generation of RNA sequences from target structures and their evaluation using *in silico* criteria. These approaches support applications in RNA therapeutics, synthetic biology, and functional genomics, and are integrated with experimental validation through iterative design-build-test cycles, linking computational predictions to biological function.

Beyond methodological advances, RNA design is increasingly being translated into practical biomedical and biotechnological applications [103]. As illustrated in Fig. 4, computational RNA design approaches provide a unifying conceptual framework that connects inverse folding algorithms with downstream applications in RNA therapeutics, synthetic biology, and functional genomics. These approaches enable the rational design of RNA molecules with desired structural and functional properties, followed by computational evaluation and iterative experimental validation. Such design-build-test cycles are becoming central to modern RNA engineering, highlighting the growing integration of computational methods with experimental and translational research.

## Concluding remarks

There are a variety of RNA design programs that were developed in the past decade. Mostly, these programs are for specific uses that emerged. Moreover, many established programs are being improved with updated version. Artificial intelligence has gained popularity for RNA design tasks as well, although in the realm of RNA secondary structure, it is only for certain problems (albeit with biotechnological importance) such as mRNA design and CRISPR gRNA design that there could be a meaningful advantage in using ML/AI based methods (encouragingly with added components, such as in [94, 96]), due to the problematic data on which they are trained. Advanced computational methodologies beyond ML/AI are still considered of utmost importance in rational RNA design, as in [48], where the Infrared framework was employed. There is a variety of leading programs that are well maintained and offer user-friendly platforms with easy-to-follow documentation, which are notably helpful for practitioners. Other programs should follow suit. This way, practitioners can continue to have a wide enough selection to choose which program is more suitable for their needs according to the capabilities and the specific types of constraints that are available in each one of the programs.

**Key Points**

- In the last decade, many new RNA design programs have been developed, while past methodologies still remain cornerstone.
- Although machine learning models are most popular in recent years, for the inverse RNA folding from secondary structure to sequences, biophysical models are still most important because of non-ideal training data that presents a key limitation.
- Programs for mRNA design are rapidly evolving, mostly also using traditional RNA design principles that should be acknowledged. RNA design on all fronts remains important for a wide range of applications, and general computational frameworks have been developed to handle a variety of constraints and complex design objectives.

## Data Availability

The data underlying this article are available in the article.

## References

[1] Hofacker IL, Fontana W, Stadler PF et al. Fast folding and comparison of RNA secondary structures. *Monatsh Chem* 1994;125:167–88. 10.1007/BF00818163

[2] Taft RJ, Pang KC, Mercer TR et al. Non-coding RNAs: regulators of disease. *J Pathol* 2010;220:126–39. 10.1002/path.2638 19882673

[3] Hammann C, Westhof E. Searching genomes for ribozymes and riboswitches. *Genome Biol* 2007;8:210. 10.1186/gb-2007-8-4-210 17472738 PMC1895996

[4] Strobel SA, Cochrane JC. RNA catalysis: ribozymes, ribosomes, and riboswitches. *Curr Opin Chem Biol* 2007;11:636–43. 10.1016/j.cbpa.2007.09.010 17981494 PMC2184879

[5] Breaker RR . Prospects for riboswitch discovery and analysis. *Mol Cell* 2011;43:867–79. 10.1016/j.molcel.2011.08.024 21925376 PMC4140403

[6] Serganov A, Nudler E. A decade of riboswitches. *Cell* 2013;152:17–24. 10.1016/j.cell.2012.12.024 23332744 PMC4215550

[7] Isaacs FJ, Dwyer DJ, Collins JJ. RNA synthetic biology. *Nat Biotechnol* 2006;24:545–54. 10.1038/nbt1208 16680139

[8] Chappell J, Watters KE, Takahashi MK et al. A renaissance in RNA synthetic biology: new mechanisms, applications and tools for the future. *Curr Opin Chem Biol* 2015;28:47–56. 10.1016/j.cbpa.2015.05.018 26093826

[9] Jaeger L, Westhof E, Leontis NB. TectoRNA: modular assembly units for the construction of RNA nano-objects. *Nucleic Acids Res* 2001;29:455–63. 10.1093/nar/29.2.455 11139616 PMC29663

[10] Cohen B, Skiena S. Natural selection and algorithmic design of mRNA. *J Comp Biol* 2003;10:419–32. 10.1089/10665270360688101 12935336

[11] Mueller S, Coleman JR, Papamichail D et al. Live attenuated influenza virus vaccines by computer-aided rational design. *Nature Biotechnol* 2010;28:723–6. 10.1038/nbt.1636 20543832 PMC2902615

[12] Bindewald E, Afonin K, Jaeger L et al. Multi-stranded RNA secondary structure prediction and nanostructure design including pseudoknots. *ACS Nano* 2011;5:9542–51. 10.1021/nn202666w 22067111 PMC3263976

[13] Findeiß S, Wachsmuth M, Mörl M et al. Design of transcription regulating riboswitches. *Methods Enzymol* 2015;550:1–22. 10.1016/bs.mie.2014.10.029 25605378

[14] Xiang X, Corsi GI, Anthon C et al. Enhancing CRISPR-Cas9 gRNA efficiency prediction by data integration and deep learning. *Nat Commun* 2021;12:3238. 10.1038/s41467-021-23576-0 34050182 PMC8163799

[15] Schuster P, Fontana W, Stadler PF et al. From sequences to shapes and back: a case study in RNA secondary structures. *Proc Biol Sci* 1994;255:279–84. 7517565 10.1098/rspb.1994.0040

[16] Hofacker IL . Vienna RNA secondary structure server. *Nucleic Acids Res* 2003;31:3429–31. 10.1093/nar/gkg599 12824340 PMC169005

[17] Lorenz R, Bernhart SH, Höner Zu Siederdissen C et al. ViennaRNA package 2.0. *Algorithms Mol Biol* 2011;8:938–46. 10.1186/1748-7188-6-26 PMC3319429 22115189

[18] Zuker M . Mfold web server for nucleic acid folding and hybridization prediction. *Nucleic Acids Res* 2003;31:3406–15. 10.1093/nar/gkg595 12824337 PMC169194

[19] Markham NR, Zuker M. UNAFold: software for nucleic acid folding and hybridization. *Methods Mol Biol* 2008;453:3–31. 10.1007/978-1-60327-429-6_1 18712296

[20] Mathews DH . RNA secondary structure analysis using RNAstructure. *Curr Protoc Bioinformatics* 2014;46:12.4.1–22. 10.1002/0471250953.bi1206s46 24939127

[21] Mathews DH, Sabina J, Zuker M et al. Expanded sequence dependence of thermodynamic parameters provides improved prediction of RNA secondary structure. *J Mol Biol* 1999;288:911–40. 10.1006/jmbi.1999.2700 10329189

[22] Mathews DH, Disney MD, Childs JL et al. Incorporating chemical modification constraints into a dynamic programming algorithm for prediction of RNA secondary structure. *Proc Natl Acad Sci USA* 2004;101:7287–92. 10.1073/pnas.0401799101 15123812 PMC409911

[23] Sato K, Hamada M, Asai K et al. CentroidFold: a webserver for RNA secondary structure prediction. *Nucleic Acids Res* 2009;37:W277–80. 10.1093/nar/gkp367 19435882 PMC2703931

[24] Flamm C, Wielach J, Wolfinger MT et al. Caveats to deep learning approaches to RNA secondary structure prediction. *Front Bioinform* 2022;2:835422. 10.3389/fbinf.2022.835422 36304289 PMC9580944

[25] Schneider B, Sweeny BA, Bateman A et al. When will RNA get its AlphaFold moment? *Nucleic Acids Res* 2023;51:9522–32. 10.1093/nar/gkad726 37702120 PMC10570031

[26] Busch A, Backofen R. INFO-RNA-a fast approach to inverse RNA folding. *Bioinformatics* 2006;22:1823–31. 10.1093/bioinformatics/btl194 16709587

[27] Andronescu M, Fejes AP, Hutter F et al. A new algorithm for RNA secondary structure design. *J Mol Biol* 2004;336:607–24. 10.1016/j.jmb.2003.12.041 15095976

[28] Dirks RM, Lin M, Winfree E et al. Paradigms for computational nucleic acid design. *Nucleic Acids Res* 2004;32:1392–403. 10.1093/nar/gkh291 14990744 PMC390280

[29] Zadeh JN, Wolfe BR, Pierce NA. Nucleic acid sequence design via efficient ensemble defect optimization. *J Comput Chem* 2011;32:439–52. 10.1002/jcc.21633 20717905

[30] Dromi N, Avihoo A, Barash D. Reconstruction of natural RNA sequences from RNA shape, thermodynamic stability, mutational robustness, and linguistic complexity by evolutionary computation. *J Biomol Struct Dyn* 2008;26:147–61. 10.1080/07391102.2008.10507231 18533734

[31] Taneda A . Multi-objective genetic algorithm for pseudoknotted RNA sequence design. *Front Genet* 2012;3:36. 10.3389/fgene.2012.00036 22558001 PMC3337422

[32] Garcia-Martin JA, Dotu I, Clote P. RNAiFold 2.0: a web server and software to design custom and Rfam-based RNA molecules. *Nucleic Acids Res* 2015;43:W513–21. 10.1093/nar/gkv460 26019176 PMC4489274

[33] Kleinkauf R, Mann M, Backofen R. AntaRNA: ant colony-based RNA sequence design. *Bioinformatics* 2015;31:3114–21. 10.1093/bioinformatics/btv319 26023105 PMC4576691

[34] Reinharz V, Ponty Y, Waldispühl J. A weighted sampling algorithm for the design of RNA sequences with targeted secondary structure and nucleotides distribution. *Bioinformatics* 2013;29:i308–15. 10.1093/bioinformatics/btt217 23812999 PMC3694657

[35] Weinbrand L, Avihoo A, Barash D. RNAfbinv: an interactive java application for fragment-based design of RNA sequences. *Bioinformatics* 2013;29:2938–40. 10.1093/bioinformatics/btt494 23975763

[36] Drory Retwitzer M, Reinharz V, Ponty Y et al. incaRNAfbinv: a webserver for the fragment-based design of RNA sequences. *Nucleic Acids Res* 2016;44:W308–14. 10.1093/nar/gkw440 27185893 PMC5741205

[37] Churkin A, Drory-Retwitzer M, Reinharz V et al. Design of RNAs: comparing programs for inverse RNA folding. *Brief Bioinform* 2017;19:bbw120–358. 10.1093/bib/bbw120 PMC6018860 28049135

[38] Höner Zu Siederdissen C, Hammer S, Abfalter I et al. Computational design of RNAs with complex energy landscapes. *Biopolymers* 2013;99:1124–36. 10.1002/bip.22337 23818234

[39] Lee J, Kladwang W, Lee M et al. RNA design rules from a massive open laboratory. *Proc Natl Acad Sci USA* 111:2122–7. 10.1073/pnas.1313039111 PMC3926058 24469816

[40] Koodli RV, Keep B, Coppess KR et al. EternaBrain: automated RNA design through move sets and strategies from an internet-scale RNA videogame. *PLoS Comput Biol* 2019;15:e1007059. 10.1371/journal.pcbi.1007059 31247029 PMC6597038

[41] Yesselman J, Das R. RNA-redesign: a web-server for fixed backbone 3D design of RNA. *Nucleic Acids Res* 2015;43:W498–501. 10.1093/nar/gkv465 25964298 PMC4489241

[42] Yesselman J, Eiler D, Carlson ED et al. Computational design of three-dimensional RNA structure and function. *Nat Nanotechnol* 2019;14:866–73. 10.1038/s41565-019-0517-8 31427748 PMC7324284

[43] Joshi CK, Liò P. gRNAde: a geometric deep learning pipeline for 3D RNA inverse design. *Methods Mol Biol* 2025;2847:121–35. 10.1007/978-1-0716-4079-1_8 39312140

[44] Tan C, Zhang Y, Gao Z et al. R3Design: deep tertiary structure-based RNA sequence design and beyond. *Brief Bioinform* 2025;26:bbae682. 10.1093/bib/bbae682 PMC11685104 39737572

[45] Popenda M, Szachniuk M, Antczak M et al. Automated 3D structure composition of large RNAs. *Nucleic Acids Res* 2012;40:e112. 10.1093/nar/gks339 22539264 PMC3413140

[46] Antczak M, Szachniuk M. Towards increasing the credibility of RNA design. *Methods Mol Biol* 2025;2847:137–51. 10.1007/978-1-0716-4079-1_9 39312141

[47] Boniecki MJ, Lach G, Dawson WK et al. SimRNA: a coarse-grained method for RNA folding simulations and 3D structure prediction. *Nucleic Acids Res* 2016;44:e63. 10.1093/nar/gkv1479 26687716 PMC4838351

[48] Walter J, Sidl L, Gutenbrunner K et al. Rational design of mechanically active RNAs: de novo engineering of functional exoribonuclease-resistant RNAs. *Nucleic Acids Res* 2026, 54:gkag473. 10.1093/nar/gkag473 PMC13161572 42120043

[49] Wirecki TK, Lach G, Badepally NG et al. DesiRNA: structure-based design of RNA sequences with a replica exchange Monte Carlo approach. *Nucleic Acids Res* 2025;53:gkae1306. 10.1093/nar/gkae1306 PMC11744100 39831304

[50] Waldl M, Yao H-T, Hofacker IL. Sequence design for RNA-RNA interactions. *Methods Mol Biol* 2025;2847:1–16. 10.1007/978-1-0716-4079-1_1 39312133

[51] Bonnet É, Rzążewski P, Sikora F. Designing RNA secondary structures is hard. *J Comp Biol* 2020;27:302–16. 10.1089/cmb.2019.0420 32160034

[52] Boury T, Gardelle S, Bulteau L et al. RNA inverse folding can be solved in linear time for structures without isolated stacks or base pairs. *Algorithms Mol Biol* 2025;20:20. 10.1186/s13015-025-00278-6 41137157 PMC12553252

[53] Yao H-T, Chauve C, Regnier M et al. Undesignable motifs in structural RNAs and combinatorial consequences. *J Mol Bio* 2026;92:49. 10.1007/s00285-026-02358-6 41817779

[54] Yao H-T, Ponty Y, Will S. Developing complex RNA design applications in the infrared framework. *Methods Mol Biol* 2024;2726:285–313. 10.1007/978-1-0716-3519-3_12 38780736

[55] Yao H-T, Marchand B, Berkemer SJ et al. Infrared: a declarative tree decomposition-powered framework for bioinformatics. *Algorithms Mol Biol* 2024;19:13. 10.1186/s13015-024-00258-2 38493130 PMC10943887

[56] Hammer S, Tschiatschek B, Flamm C et al. RNAblueprint: flexible multiple target nucleic acid sequence design. *Bioinformatics* 2017;33:2850–8. 10.1093/bioinformatics/btx263 28449031 PMC5870862

[57] Hammer S, Wang W, Will S et al. Fixed-parameter tractable sampling for RNA design with multiple target structures. *BMC Bioinformatics* 2019;20:209. 10.1186/s12859-019-2784-7 31023239 PMC6482512

[58] Ponty Y, Hammer S, Yao H-T et al. Advanced design of structural RNAs using RNARedPrint. *Methods Mol Biol* 2021;2284:1–15. 10.1007/978-1-0716-1307-8_1 33835434

[59] Zhu M, Mathews D. Sequence design using RNAstructure. *Methods Mol Biol* 2025;2847:17–31. 10.1007/978-1-0716-4079-1_2 39312134

[60] Fornace ME, Huang J, Newman CT et al. NUPACK: Computational nucleic acid analysis and design. ACS Synth Biol 2026; 15:1426–1441. 10.1021/acssynbio.5c00817. PMC13160402 41926704

[61] Minuesa G, Alsina C, Garcia-Martin JA et al. MoiRNAiFold: a novel tool for complex *in silico* RNA design. *Nucleic Acids Res* 2021;49:4934–43. 10.1093/nar/gkab331 33956139 PMC8136780

[62] Alsina C, Dotu I. Complex *in-silico* RNA design with MoiRNAiFold. *Methods Mol Biol* 2025;2847:45–61. 10.1007/978-1-0716-4079-1_4 39312136

[63] Drory-Retwizer M, Reinharz V, Churkin A et al. incaRNAfbinv 2.0: a webserver and software with motif control for fragment-based design of RNAs. *Bioinformatics* 2020;36:2920–2. 10.1093/bioinformatics/btaa039 31971575

[64] Taneda A . Riboswitch design using MODENA. *Methods Mol Biol* 2025;2847:33–43. 10.1007/978-1-0716-4079-1_3 39312135

[65] Tsang HH . Simulated annealing for RNA design with SIMARD. *Methods Mol Biol* 2025;2847:95–108. 10.1007/978-1-0716-4079-1_6 39312138

[66] Badura J, Rybarczyk A, Zok T. Comprehensive datasets for RNA design, machine learning, and beyond. *Sci Rep* 2025;15:21417. 10.1038/s41598-025-07041-2 40594473 PMC12215848

[67] Shor J, Strand E, McLean CY. NucleoBench: a large-scale benchmark of neural nucleic acid design algorithms. *bioRxiv* 2025:1–16. 2025.06.20.660785

[68] T. Cazenave Eterna is solved. *arXiv* 2025: 2505.02110.

[69] Cazenave T, Touzani H. Monte Carlo inverse RNA folding. *Methods Mol Biol* 2025;2847:205–15. 10.1007/978-1-0716-4079-1_14 39312146

[70] Ontiveros-Palacios N, Cooke E, Nawrocki EP et al. Rfam 15: RNA families database in 2025. *Nucleic Acids Res* 2025;53:D258–67. 10.1093/nar/gkae1023 39526405 PMC11701678

[71] Mukherjee S, Mandal SD, Gupta N et al. RiboD: a comprehensive database for prokaryotic riboswitches. *Bioinformatics* 2019;35:3541–3. 10.1093/bioinformatics/btz093 30726866

[72] Brandenburg VB, Narberhaus F, Mosig A. Inverse folding based pre-training for the reliable identification of intrinsic transcription terminators. *PLoS Comput Biol* 2022;18:e1010240. 10.1371/journal.pcbi.1010240 35797361 PMC9262186

[73] Dotu I, Lozano G, Clote P et al. Using RNA inverse folding to identify IRES-like structural subdomains. *RNA Biol* 2013;10:1842–52. 10.4161/rna.26994 24253111 PMC3917987

[74] Yamagami R, Kayedkhordeh M, Mathews DH et al. Design of highly active double-pseudoknotted ribozymes: a combined computational and experimental study. *Nucleic Acids Res* 47:29–42. 30462314 10.1093/nar/gky1118 PMC6326823

[75] Nawrocki EP, Eddy SR. Infernal 1.1: 100-fold faster RNA homology searches. *Bioinformatics* 2013;29:2933–5. 10.1093/bioinformatics/btt509 24008419 PMC3810854

[76] Mukherjee S, Drory-Retwitzer N, Hubbell S et al. A computational approach for the identification of distant homologs of bacterial riboswitches based on inverse RNA folding. *Brief Bioinform* 2023;24:1–12. 10.1093/bib/bbad110 36951499

[77] Zakh R, Churkin A, Barash D. RNA design using incaRNAfbinv demonstrated with the identification of functional RNA motifs in hepatitis delta virus. *Methods Mol Biol* 2025;2847:109–20. 10.1007/978-1-0716-4079-1_7 39312139

[78] Zakh R, Churkin A, Parr M et al. The bioinformatics of the finding that the hepatitis delta virus RNA editing mechanism by a conformational switch exists in genotype 7 in addition to genotype 3. *Brief Bioinform* 2025;26:bbae451. 10.1093/bib/bbaf451 PMC12406691 40900114

[79] Runge F, Hutter F. Machine learning for RNA design: LEARNA. *Methods Mol Biol* 2025;2847:63–93. 10.1007/978-1-0716-4079-1_5 39312137

[80] Li Y, Pan X, Shen H et al. DRAG: design RNAs as hierarchical graphs with reinforcement learning. *Brief Bioinform* 2025;26:bbaf106. 10.1093/bib/bbaf106 PMC11904406 40079262

[81] Anthon C, Corsi GI, Gorodkin J. CRISPRon/off: CRISPR/Cas9 on- and off-target gRNA design. *Bioinformatics* 2022;38:5437–9. 10.1093/bioinformatics/btac697 36271848 PMC9750111

[82] Sun Y, Qu K, Corsi GI et al. Deep learning models simultaneously trained on multiple datasets improve base-editing activity prediction. *Nat Commun* 2025;16:9821. 10.1038/s41467-025-65200-5 41203686 PMC12595020

[83] Chakraborty S, Minary P. The evolution of nucleic acid-based diagnosis methods from the (pre-)CRISPR to CRISPR era and the associated machine/deep leaning approaches in relevant RNA design. *Methods Mol Biol* 2025;2847:241–300. 10.1007/978-1-0716-4079-1_17 39312149

[84] Fernandez-de-Cossio-Diaz J, Hardouin P, Lyonnet du Moutier F-X et al. Designing molecular RNA switches with restricted Boltzmann machines. *Nat Commun* 2025;16:11223. 10.1038/s41467-025-66265-y 41413030 PMC12714719

[85] Iwano N, Adachi T, Aoki K et al. Generative aptamer discovery using RaptGen. *Nat Comput Sci* 2022;2:378–86. 10.1038/s43588-022-00249-6 38177576 PMC10766510

[86] Sumi S, Hamada M, Saito H. Deep generative design of RNA family sequences. *Nat Meth* 2024;21:435–43. 10.1038/s41592-023-02148-8 38238559

[87] Hamada M . RNAdesign. *Drug Delivery Sys* 2024;39:333–45. 10.2745/dds.39.333

[88] A Churkin, D Barash. (eds.) RNA Design: Methods and Protocols. New York: Springer, 2025, 10.1007/978-1-0716-4079-1.

[89] Ward M, Richardson M, Metkar M. mRNA folding algorithms for structure and codon optimization. *Brief Bioinform* 2025;26:bbaf386. 10.1093/bib/bbaf386 40755283 PMC12319323

[90] Zhang H, Zhang L, Ang L et al. Algorithm for optimized mRNA design improves stability and immunogenicity. *Nature* 2023;621:396–403. 10.1038/s41586-023-06127-z 37130545 PMC10499610

[91] Tang WY, Dai N, Zhou T et al. SamplingDesign: RNA design via continuous optimization with coupled variables and Monte-Carlo sampling. *Nat Commun* 2026; 17:2950. 10.1038/s41467-025-67901-3 PMC13031908 41714588

[92] Krueger RK, Ward M. JAX-RNAfold: scalable differentiable folding. *Bioinformatics* 2025;41:btaf203. 10.1093/bioinformatics/btaf203 40279486 PMC12064173

[93] Matthies MC, Krueger R, Torda AE et al. Differentiable partition function calculation for RNA. *Nucleic Acids Res* 2024;52:e14. 10.1093/nar/gkad1168 38038257 PMC10853804

[94] Dai N, Zhou T, Tang WY et al. EnsembleDesign: messenger RNA design minimizing ensemble free energy via probabilistic lattice parsing. *Bioinformatics* 2025;41:i391–400. 10.1093/bioinformatics/btaf245 40662826 PMC12261492

[95] Li S, Noroozizadeh S, Moayedpour S et al. mRNA-LM: full-length integrated SLM for mRNA analysis. *Nucleic Acids Res* 2025;53:gkaf044. 10.1093/nar/gkaf044 PMC11962594 39898548

[96] Li S, Chauvin P, Gross O et al. mRNA-GPT: end-to-end generative design and optimization of full-length mRNA ICLR 2026 Workshop on Generative and Experimental Perspectives for Biomolecular Design.

[97] Zhang H, Liu H, Xu Y et al. Deep generative models design mRNA sequences with enhanced translational capacity and stability. *Science* 2025;390:eadr8470. 10.1126/science.adr8470 40875799

[98] Mo O, Zhang Z, Cheng X et al. mRNAdesigner: an integrated web server for optimizing mRNA design and protein translation in eukaryotes. *Nucleic Acids Res* 2025;53:W415–26. 10.1093/nar/gkaf410 40384581 PMC12230703

[99] Budzinska MA, Zardbani M, Michelle PS et al. mRNArchitect: sequence design of mRNA medicines. *bioRxiv* 2025:1–11. 2024.12.03.626696

[100] Riley AT, Robson JM, Ulanova A et al. Generative and predictive neural networks for the design of functional RNA molecules. *Nat Commun* 2025;16:4155. 10.1038/s41467-025-59389-8 40320400 PMC12050331

[101] Leppek K, Byeon GW, Kladwang W et al. Combinatorial optimization of mRNA structure, stability, and translation for RNA-based therapeutics. *Nat Commun* 2022;13:1536. 10.1038/s41467-022-28776-w 35318324 PMC8940940

[102] Waymant-Steele HK, Kim DS, Choe CA et al. Theoretical basis for stabilizing messenger RNA through secondary structure design. *Nucleic Acids Res* 2021;49:10604–17. 10.1093/nar/gkab764 34520542 PMC8499941

[103] Mukherjee S, Barash D. Designing RNA switches for synthetic biology using inverse RNA folding. *Trends Biotechnol* 2025;42:517–21. 10.1016/j.tibtech.2023.11.005 38040620

